# Mixture effects of prenatal exposure to per- and polyfluoroalkyl substances and polybrominated diphenyl ethers on maternal and newborn telomere length

**DOI:** 10.1186/s12940-021-00765-4

**Published:** 2021-06-30

**Authors:** Stephanie M. Eick, Dana E. Goin, Lara Cushing, Erin DeMicco, June-Soo Park, Yunzhu Wang, Sabrina Smith, Amy M. Padula, Tracey J. Woodruff, Rachel Morello-Frosch

**Affiliations:** 1 Program On Reproductive Health and the Environment, Department of Obstetrics, Gynecology and Reproductive Sciences, University of California, San Francisco, USA; 2 Department of Environmental Health Sciences, Fielding School of Public Health, University of California, Los Angeles, USA; 3 Environmental Chemistry Laboratory, Department of Toxic Substances Control, California Environmental Protection Agency, Berkeley, USA; 4 Department of Environmental Science, Policy and Management and School of Public Health, University of California, Berkeley, USA

**Keywords:** Per- and poly-fluoroalkyl substances, Polybrominated diphenyl ethers, Telomere, Exposure mixture

## Abstract

**Background:**

Per- and polyfluoroalkyl substances (PFAS) and polybrominated diphenyl ethers (PBDEs) are endocrine disrupting chemicals with widespread exposures across the U.S. given their abundance in consumer products. PFAS and PBDEs are associated with reproductive toxicity and adverse health outcomes, including certain cancers. PFAS and PBDEs may affect health through alternations in telomere length. In this study, we examined joint associations between prenatal exposure to PFAS, PBDEs, and maternal and newborn telomere length using mixture analyses, to characterize effects of cumulative environmental chemical exposures.

**Methods:**

Study participants were enrolled in the Chemicals in Our Bodies (CIOB) study, a demographically diverse cohort of pregnant people and children in San Francisco, CA. Seven PFAS (ng/mL) and four PBDEs (ng/g lipid) were measured in second trimester maternal serum samples. Telomere length (T/S ratio) was measured in delivery cord blood of 292 newborns and 110 second trimester maternal whole blood samples. Quantile g-computation was used to assess the joint associations between groups of PFAS and PBDEs and newborn and maternal telomere length. Groups considered were: (1) all PFAS and PBDEs combined, (2) PFAS, and (3) PBDEs. Maternal and newborn telomere length were modeled as separate outcomes.

**Results:**

T/S ratios in newborn cord and maternal whole blood were moderately correlated (Spearman ρ = 0.31). In mixtures analyses, a simultaneous one quartile increase in all PFAS and PBDEs was associated with a small increase in newborn (mean change per quartile increase = 0.03, 95% confidence interval [CI] = -0.03, 0.08) and maternal telomere length (mean change per quartile increase = 0.03 (95% CI = -0.03, 0.09). When restricted to maternal–fetal paired samples (*N *= 76), increasing all PFAS and PBDEs combined was associated with a strong, positive increase in newborn telomere length (mean change per quartile increase = 0.16, 95% CI = 0.03, 0.28). These associations were primarily driven by PFAS (mean change per quartile increase = 0.11 [95% CI = 0.01, 0.22]). No associations were observed with maternal telomere length among paired samples.

**Conclusions:**

Our findings suggest that PFAS and PBDEs may be positively associated with newborn telomere length.

**Supplementary Information:**

The online version contains supplementary material available at 10.1186/s12940-021-00765-4.

## Introduction

Exposure to per- and polyfluoroalkyl substances (PFAS) and polybrominated diphenyl ethers (PBDEs) is widespread in the U.S. given their abundant use in consumer products and persistence in the environment [1]. PFAS and PBDEs are classified as human carcinogens and exposure has been linked to numerous adverse health outcomes including certain cancers, thyroid disease, and developmental delay in children [1, 2]. Prenatal exposure to PFAS and PBDEs is particularly concerning, as these chemicals have been detected 99% of pregnant women [3], in cord blood [4] and the placenta [5, 6] and are linked to adverse reproductive outcomes, such as preterm birth and low birth weight [6–8]. Despite their ubiquity and toxicity, the biologic pathways linking PFAS and PBDEs to adverse outcomes remain poorly understood.

It is possible that PFAS and PBDEs influence adverse health outcomes through alterations in telomere length. Telomeres are the specific DNA–protein structures located at the end of each chromosome that protect chromosomes from degradation [9]. Telomere shortening occurs at each DNA replication, thus telomeres are longest at birth and decrease across the life course [10]. Telomere length at birth may be a predictor of disease risk later in life, as newborn telomere length is predictive of telomere length in adulthood [11]. Among adults, shorter telomeres have been associated with certain chronic and age-related conditions such as cardiovascular disease, obesity, and premature death [10, 12]. In contrast, longer telomeres have been associated with certain types of cancers [13, 14]. During infancy, longer maternal telomere length is linked to impaired cognitive development in infants at 7.5 months [15]. Telomere length at birth can be influenced by a variety of factors including genetic heritability [16], prenatal stress, [17, 18] parental socioeconomic status, [19] and maternal smoking [20].

Environmental chemicals have been associated with changes in telomere length. For example, studies of mother-infant pairs in China found that increasing arsenic and manganese concentrations were associated with longer newborn telomere length [21, 22]. In contrast, prenatal lead exposure was associated with a reduction in newborn telomere length in a pregnancy cohort in Argentina [23]. Increasing concentrations of certain metals and phthalate metabolites have also been linked to shorter newborn telomere length [24, 25]. To date, the relationship between PFAS, PBDEs, and telomere length remains largely unknown. One study of 103 pregnant women in San Francisco found that prenatal exposure to PFAS or PBDEs was not associated with newborn telomere length [26]. In contrast, Perfluorooctanoic Acid (PFOA) and Perfluorohexanesulphonic Acid (PFHxS) have been linked to telomere shortening among adults ages 50–65 [27]. Perflucorooctane Sulfonic Acid (PFOS) has also been associated with telomere lengthening among adults in the U.S. and Belgium [27, 28].

While several studies have examined the effects of environmental chemicals on newborn telomere length, few studies have looked at associations between environmental chemicals and maternal telomere length during pregnancy. This may be particularly important, as prior work has shown that certain metals affect maternal and newborn telomere length in opposing directions [23]. Furthermore, most prior work has assessed the effect of chemicals individually. Given that exposures to many of these chemicals are highly correlated and may influence similar biological mechanisms, considering chemical exposures as mixtures may improve our understanding of the effects of environmental chemicals on telomere length. Therefore, in the present study, we utilized quantile g-computation [29], a novel method for the analysis of exposure mixtures, to examine the joint effects of prenatal exposure to PFAS and PBDEs on maternal and newborn telomere length in a demographically diverse cohort pregnant people in San Francisco.

## Methods

### Study population

Participants included in the present analysis were a subset of participants from the Chemicals in Our Bodies (CIOB) cohort, which has been previously described in detail elsewhere [30]. CIOB is an ongoing, prospective pregnancy cohort designed to examine the cumulative effects of environmental chemicals and psychosocial stressors on fetal growth and child development. Since 2014, pregnant women have been recruited during their second trimester from the Moffit Long, Mission Bay, and Zuckerberg San Francisco General Hospitals in San Francisco, CA. Inclusion criteria for CIOB include: ≥ 18 years of age, English or Spanish speaking, and not pregnant with multiples. All participants provided written, informed consent prior to participating and the Institutional Review Boards at the University of California, San Francisco (UCSF) and Berkeley approved this study (Protocol # 13–12160). There were 509 participants enrolled in the CIOB study. Information on PFAS and PBDE levels were available in all participants and a subset of participants had whole blood samples collected and analyzed for maternal (*N *= 125) and newborn (*N *= 326) telomere length. We conducted a complete case analysis and excluded 37 participants who had information on either maternal or newborn telomere length but were missing any of the following covariates: pre-pregnancy body mass index (BMI), education, race/ethnicity, or parity. Our final analytic sample included 292 participants with information on newborn telomere length, 110 participants with information on maternal telomere length, and there were 76 maternal–fetal unit matched pairs.

### Per- and poly-fluoroalkyl substances and polybrominated diphenyl ethers

Maternal whole blood samples were obtained during the second trimester, between 12 and 28 weeks gestation, in red top tubes. Serum samples were frozen at -80 °C for analysis of 12 PFAS and 19 PBDEs at the Environmental Chemical Laboratory at the California Department of Toxic Substances Control (DTSC). We focused our analysis on those PFAS (ng/mL) and PBDEs (ng/g lipid) with ≥ 90% of samples having a machine read value. Using this cut point, we included 7 PFAS and 4 PBDEs in our analysis, which were: Perfluorononanoic Acid [PFNA], Perfluorooctanoic Acid [PFOA], Perfluorohexanesulphonic Acid [PFHxS], Perflucorooctane Sulfonic Acid [PFOS], Methyl-perfluorooctane sulfonamide Acetic Acid [Me-PFOSA-AcOH], Perfluorodecanoic Acid [PFDeA], Perfluoroundecanoic Acid [PFUdA], 2,2′,4,4′-tetra-bromodiphenyl ether [BDE-47], 2,2′,4,4′,5-penta-bromodiphenyl ether [BDE-99], 2,2′,4,4′,5,5′-hexa-bromodiphenyl ether [BDE-153], and 2,2′,4,4′,6-penta-bromodiphenyl ether [BDE-100]. The distribution of additional PFAS and PBDEs for which a machine read value was not available in ≥ 90% of samples is provided elsewhere [31]. PFAS were quantified by injection onto an automated on-line solid phase extraction method coupled to liquid chromatography and tandem mass spectrometry, and PBDEs were analyzed using gas chromatography/ high resolution mass spectrometry (GC-HRMS, DFS, Thermo-Scientific, Bremen, Germany) using isotope dilution. Lipid concentrations in maternal serum were also measured and wet-weight PBDEs were adjusted with lipid serum concentrations. Additional details regarding the analysis of PFAS and PBDEs is available elsewhere [31]. Measurements below the method detection limit (MDL) were assigned the machine read value if one was detected and if there was no machine read value, the concentration was replaced with MDL/$\surd$ 2. PFAS and PBDEs were right skewed and natural log transformed for analysis.

### Telomere length

Telomere length was measured at the UCSF Blackburn Lab using a well-established assay that has been described in detail elsewhere [15, 32–35]. Briefly, genomic DNA was extracted from frozen maternal whole blood and delivery cord blood with QIAamp mini DNA kit (QIAGEN, cat# 51,106). DNA quantity and quality were measured by OD260/OD280 and OD260/OD230 on a NanoDrop 2000/2000c Spectrophotometers. DNA quality criteria are OD260/OD280 between 1.7–2.0, OD260/OD230 > 1.0 and concentration > 10 ng/ul. DNA samples were extracted, stored at -80 °C and assayed as one batch. Average telomere length values were measured by a quantitative polymerase chain reaction (PCR) assay that determines the relative ratio of telomere repeat abundance to single-copy gene abundance (T/S ratio). Tubes containing 26, 8.75, 2.9, 0.97, 0.324 and 0.108 ng of a reference DNA (human genomic DNA from buffy coat, Sigma cat# 11,691,112,001) were included in each PCR run so that the quantity of targeted templates in each research sample could be determined relative to the reference DNA sample by the standard curve method. The primers for the telomere PCR were *tel1b* [5'-CGGTTT(GTTTGG)_5_GTT-3'], used at a final concentration of 100 nM, and *tel2b* [5'-GGCTTG(CCTTAC)_5_CCT-3'], used at a final concentration of 900 nM. The primers for the single-copy gene (human beta-globin) PCR were *hbg1* [5'-GCTTCTGACACAACTGTGTTCACTAGC-3'], used at a final concentration of 300 nM, and *hbg2* [5'-CACCAACTTCATCCACGTTCACC-3'], and used at a final concentration of 700 nM. All samples were measured twice with triplicate wells and the same lot of reagents was used for the telomere length assay. Lab personnel who performed the assays were provided with de-identified samples and were blind to all demographic and clinical data. The average coefficient of variation (CV) for this study was 2.3% and the intra-class correlation coefficient (ICC) was 97%. The maternal and newborn T/S ratios were normally distributed, and no transformations were applied.

### Covariates

Maternal education and race/ethnicity were self-reported using an interview questionnaire that was administered during the second trimester. Information regarding maternal age, gestational age at delivery, pre-pregnancy BMI (kg/m^2^), parity, and infant sex were abstracted from the participants’ medical record.

### Statistical analysis

We used quantile g-computation to estimate the joint effects of PFAS and PBDEs in relation to maternal and newborn telomere length. Quantile g-computation estimates the effect of simultaneously increasing all exposures in the mixture by one quartile using a parametric, generalized linear model based implementation of g-computation [29]. This method allows for individual exposure-outcome relationships in the mixture to go in opposing directions and produces unbiased estimates of overall mixture effects with appropriate confidence interval (CI) coverage in small sample sizes [29]. Quantile g-computation categorizes all individual PFAS and PBDEs into quantiles and each exposure is given a positive or negative weight. Weights in the positive and negative direction each sum to one. If the individual PFAS and PBDEs have different directions of effect, the weights are interpreted as the proportion of the partial effect in the positive or negative direction.

We examined three mixture groups in our analyses: (1) all PFAS and PBDEs combined, (2) PFAS, and (3) PBDEs. When the mixture effect for the PFAS and PBDEs subgroups were estimated, the remaining chemicals were retained in the model as covariates. Maternal age, race/ethnicity, education, parity, and pre-pregnancy BMI were additionally included as covariates in all models and were chosen via a directed acyclic graph (DAG). We did not retain information on smoking status and alcohol consumption in adjusted models as very few participants reported smoking (1%) or drinking alcohol (0.6%) during pregnancy. For all covariates, there was evidence of an association between exposures and outcomes in bivariate analyses and prior studies have suggested that these covariates are associated with environmental chemicals and telomere length [36, 37]. We chose not to adjust for gestational age at delivery in models where newborn telomere length was the outcome of interest because we hypothesized that it may mediate the relationship between PFAS, PBDEs and newborn telomere length [38]. Because newborn telomere attrition may be attributed to genetic inheritance [16], we also conducted an additional analysis that was restricted to paired maternal–fetal samples (*N *= 76).

We examined non-linearity of the effect of the overall mixture on maternal and newborn telomere length using two approaches [29, 39]. First, we altered the number of quantiles, ranging from 2 to 20, to assess whether different numbers of exposure categories would better capture non-linear effects. In the second approach, we contrasted two models: 1) all exposures were entered as linear terms and the overall estimate was assumed to be linear; 2) all exposures in the model were entered as linear and quadratic terms and the overall effect could be quadratic. In these models, confidence intervals were estimated using non-parametric bootstrapping with 300 samples and we compared the coefficients from the two models.

All PFAS and PBDEs included in our analyses had a machine read value in > 93% of samples. Given that the remaining proportion of values below the MDL was less than 1 divided by the number of quartiles in our primary analyses, all methods to handle missing data would have resulted in the same estimate as all imputed values would be in the lowest exposure category. Therefore, for simplicity, missing values where a machine read value was not detected were replaced with the MDL/$\surd$ 2.

We conducted numerous sensitivity analyses to examine the robustness of our findings. First, we additionally adjusted for nativity as prior studies have shown that PFAS and PBDE levels differ depending on whether women are born outside the U.S [40, 41]. Second, in models restricted to matched maternal–fetal paired samples, we additionally adjusted for maternal telomere length in models which included newborn telomere length as the outcome of interest. Lastly, we evaluated effect modification by infant sex using stratified quantile g-computation models.

To facilitate comparisons with prior work, we also examined individual PFAS and PBDEs in relation to maternal and newborn telomere length using linear regression models adjusted for maternal age, race/ethnicity, education, parity and pre-pregnancy BMI. In these models, beta estimates were standardized to the population’s interquartile range (IQR) to reflect the difference in telomere length associated with an IQR increase in individual PFAS and PBDEs.

## Results

In our analytic sample, a majority of participants were white or Latina and had college or graduate degrees (Table 1). Approximately half of study participants had one or more prior births and mean gestational age at delivery was 39 weeks. The distribution of demographics and PFAS and PBDE exposure was similar of the larger CIOB cohort (Table S1) [30]. When restricting to maternal–fetal paired samples, participants were more likely to have a college or graduate degree and be white relative to the larger study population (Table 1).

**Table 1:** Description of Chemicals in Our Bodies analytic sample analyzing per- and poly-fluoroalkyl substances, and polybrominated diphenyl ethers and maternal and newborn telomere length

	**Newborn Subsample (***N ***= 292)**	**Maternal Subsample (*N *= 110)**	**Matched Maternal–Fetal Unit Pairs Subsample (*N *= 76)**
	**N (%) or Mean (SD)**	**N (%) or Mean (SD)**	**N (%) or Mean (SD)**
Maternal Age at Enrollment, years			
Mean (SD)	34 (5.0)	33 (5.4)	34 (5.1)
Gestational age at delivery			
Mean (SD)	39 (1.7)	39 (1.5)	39 (1.7)
Pre-pregnancy BMI (kg/m^2^)			
Mean (SD)	25 (5.1)	26 (5.6)	25 (5.2)
Maternal Education			
Less than High School	25 (9%)	14 (13%)	4 (5%)
High School Degree or Some College	59 (20%)	24 (22%)	11 (14%)
College Degree	81 (28%)	34 (31%)	28 (37%)
Graduate Degree	127 (43%)	38 (35%)	33 (43%)
Maternal Race/Ethnicity			
Non-Hispanic White	126 (43%)	46 (42%)	40 (53%)
Non-Hispanic Black	10 (3%)	4 (4%)	2 (3%)
Asian/Pacific Islander	62 (21%)	16 (15%)	14 (18%)
Latina	85 (29%)	39 (35%)	17 (22%)
Other	9 (3%)	5 (5%)	3 (4%)
Parity			
No Prior Births	162 (55%)	52 (47%)	40 (53%)
One or More Prior Births	130 (45%)	58 (53%)	36 (47%)

*Abbreviations*: *SD* Standard deviation

Note: There are 326 pregnant participants, of which there are 76 matched maternal–fetal unit pairs

**Fig. 1:**
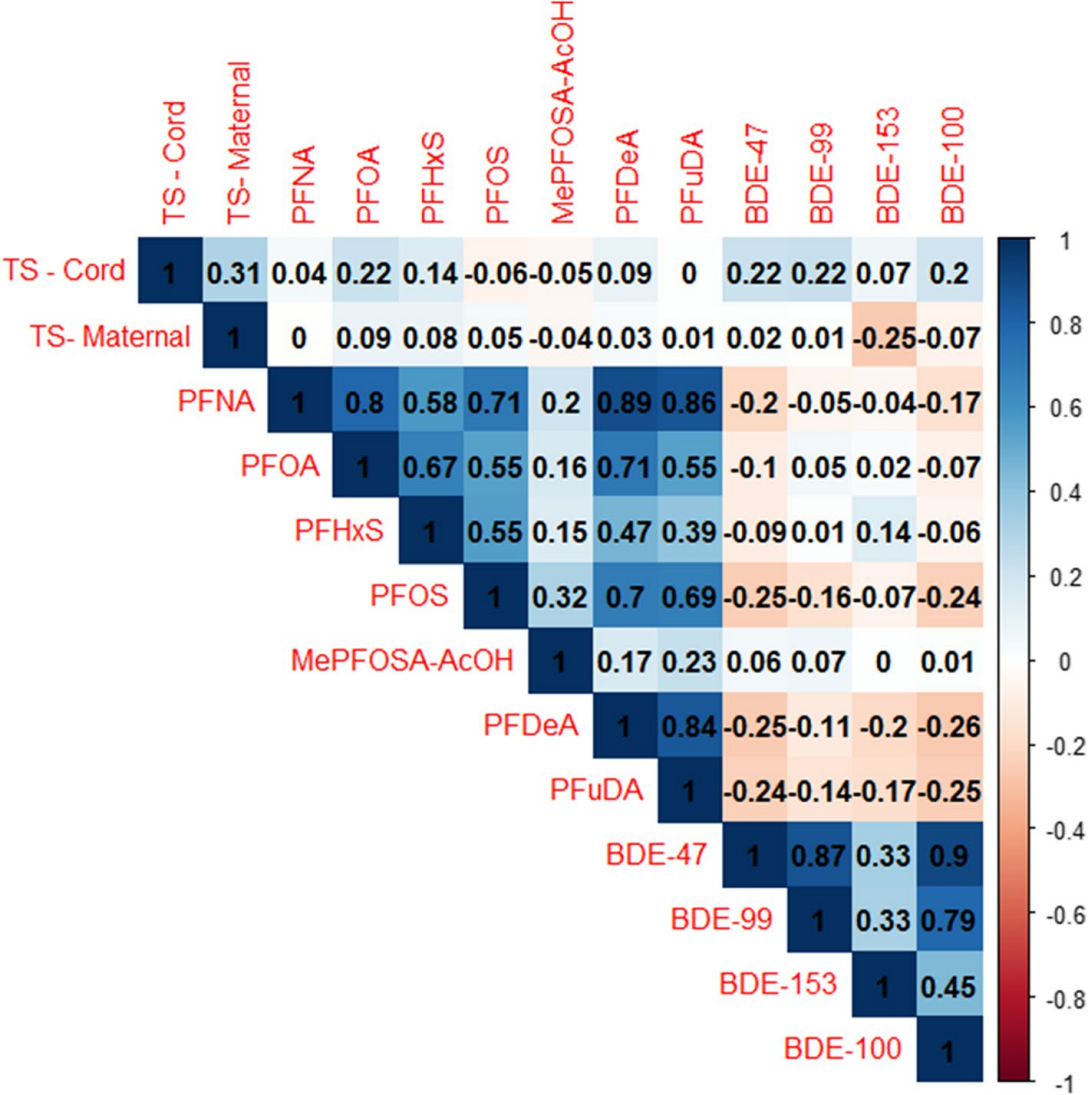
Spearman correlations between telomere length (T/S ratio), per- and poly-fluoroalkyl substances (ng/mL), and polybrominated diphenyl ethers (ng/g lipid) among paired samples (*N *= 76)

The geometric mean of newborn and maternal telomere length was 1.48 (T/S ratio) and 1.06 (T/S ratio), respectively (Table 2). Among the PFAS, PFOS had the highest geometric mean (1.95 ng/mL) and among the PBDEs, BDE-47 had the highest geometric mean (10.7 ng/g lipid) (Table 2). Relative to the larger analytic sample the geometric mean of BDE-153 was slightly higher among matched paired samples (6.9 ng/g lipid versus 5.9 ng/g lipid), while the distribution of maternal and newborn telomere length and PFAS were similar (Table S2). Maternal and newborn telomere length were moderately correlated with one another (Spearman ρ = 0.31) but were weakly correlated with PFAS or PBDEs (Fig. 1). PFAS were also correlated with each other with the strongest correlation between PFDeA and PFNA (Spearman ρ = 0.89). PBDEs were moderately to strongly correlated with one another (Spearman ρ ranging from 0.33 to 0.90), but they were not correlated or were weakly inversely correlated with PFAS (Fig. 1).

**Table 2:** Distribution of telomere length (T/S ratio) in whole blood, per- and poly-fluoroalkyl substances (ng/mL), and polybrominated diphenyl ethers (ng/g lipid) in serum

	**N**	**% Above MDL**	**% Machine Readable**	**Geometric Mean**	**Geometric SD**	**Percentiles**				
						**5%**	**25%**	**50%**	**75%**	**95%**
**Telomere Length**										
Newborn	292	100	100	1.5	1.2	1.1	1.3	1.5	1.7	1.9
Maternal	110	100	100	1.1	1.2	0.9	1.0	1.1	1.2	1.3
**Per- and poly-fluoroalkyl substances (PFAS)**										
PFNA	326	98.5	99.7	0.3	2.1	0.1	0.2	0.3	0.5	0.9
PFOA	326	100	100	0.8	2.0	0.3	0.5	0.8	1.2	2.2
PFHxS	326	100	100	0.4	2.2	0.1	0.2	0.3	0.6	1.5
PFOS	326	100	100	2.0	2.1	0.6	1.2	2.0	3.2	6.1
Me-PFOSA-AcOH	326	98.5	99.7	0.1	2.2	0.0	0.0	0.1	0.1	0.2
PFDeA	326	74.2	92.3	0.1	2.3	0.0	0.1	0.1	0.2	0.5
PFUdA	326	77.3	95.1	0.1	2.6	0.0	0.1	0.1	0.2	0.4
**Polybrominated diphenyl ethers (PBDEs)**										
BDE-47	326	99.7	100	10.7	2.1	3.6	6.8	9.6	15.7	36.9
BDE-99	326	81.9	99.1	3.4	2.0	1.3	2.4	3.2	4.9	10.4
BDE-153	326	57.7	94.2	5.9	2.8	1.4	3.0	5.4	10.3	35.9
BDE-100	326	41.1	100	2.2	2.3	0.8	1.2	1.9	3.2	8.6

*Abbreviations*: *MDL* Method detection limit, *SD* Standard deviation

*Note*: Geometric mean, geometric SD, and percentile values use the machine read value if it was available. If there was no machine read value, it was replaced with MDL/square root of 2. There are 326 pregnant participants, of which there are 76 matched maternal–fetal unit pairs

Using quantile g-computation, increasing all PFAS and PBDEs in the mixture by one quartile was associated with a small increase newborn telomere length (mean change in newborn telomere length per quartile increase = 0.03, 95% CI = -0.03, 0.08) (Table 3). Similarly, when examining PBDEs alone, we observed a positive association with newborn telomere length (mean change in newborn telomere length per quartile increase = 0.03, 95% CI = -0.01, 0.06). In this model, BDE-47 and BDE-99 were assigned the largest positive weights (Figure S1). When examining PFAS alone, we observed a negligible association (mean change in newborn telomere length per quartile increase = 0.00, 95% CI = -0.04, 0.05). The overall PFAS and PBDEs mixture was similarly associated with a slight increase maternal telomere length (mean change in maternal telomere length per quartile increase = 0.03, 95% CI = -0.03, 0.09). The PBDE mixture alone was associated with a small reduction in maternal telomere length (mean change in maternal telomere length per quartile increase = -0.02, 95% CI = -0.06, 0.02) and BDE-153 was assigned the largest negative weight (Figure S2). The PFAS mixture alone was associated with a modest increase in maternal telomere length (mean change in maternal telomere length per quartile increase = 0.04, 95% CI = -0.01, 0.09). Additional adjustment for nativity did not substantially change effect estimates (Table S3). Furthermore, altering the number of quartiles used to characterize the overall PFAS and PBDEs mixture did not noticeably change our results (Table S4) and no evidence of non-linearity was observed when the overall mixture was allowed to be quadratic (data not shown).

**Table 3:** Quantile g-computation estimates and 95% confidence intervals for the change in newborn and maternal telomere length (T/S ratio) for a one quartile increase within the (a) overall mixture of PFAS and PBDEs, (b) PFAS and (c) PBDEs in all samples and among paired samples

		**β**	**95% CI**
**All Samples**			
**Newborn Telomere Length (***N ***= 292)**			
Overall^a^		0.03	(-0.03, 0.08)
PFAS^b^		0.00	(-0.04, 0.05)
PBDEs^c^		0.03	(-0.01, 0.06)
**Maternal Telomere Length (***N ***= 110)**			
Overall^a^		0.03	(-0.03, 0.09)
PFAS^b^		0.04	(-0.01, 0.09)
PBDEs^c^		-0.02	(-0.06, 0.02)
**Matched Maternal–Fetal Unit Paired Samples**			
**Newborn Telomere Length (***N ***= 76)**			
Overall^a^		0.16	(0.03, 0.28)
PFAS^b^		0.11	(0.01, 0.22)
PBDEs^c^		0.07	(-0.01, 0.15)
**Maternal Telomere Length (***N ***= 76)**			
Overall^a^		0.02	(-0.05, 0.10)
PFAS^b^		0.03	(-0.03, 0.10)
PBDEs^c^		-0.02	(-0.07, 0.03)

*Abbreviations*: *CI* Confidence interval

*Note*: Beta estimates are interpreted as the effect on telomere length of increasing every exposure in the mixture by one quantile. There are 326 pregnant participants, of which there are 76 matched maternal–fetal unit pairs

^a^Adjusted for maternal age, race/ethnicity, education, parity, and pre-pregnancy body mass index

^b^Mixture effect is for only PFAS, adjusted for maternal age, race/ethnicity, education, parity, pre-pregnancy body mass index, and PBDEs

^c^Mixture effect is for only PBDEs, adjusted for maternal age, race/ethnicity, education, parity, pre-pregnancy body mass index, and PFAS

In our analysis restricted to maternal–fetal unit paired samples, we observed a stronger positive association with newborn telomere length when increasing all PFAS and PBDEs by one quartile (mean change in newborn telomere length per quartile increase = 0.16, 95% CI = 0.03, 0.28) (Table 3). In contrast to our overall results, increasing all PFAS by one quartile was positively associated with newborn telomere length (mean change in newborn telomere length per quartile increase = 0.11, 95% CI = 0.01, 0.22). PFOA and PFUdA were assigned the largest positive weight for both the overall and PFAS only quantile g-computation models. PFOS and PFNA were the only chemicals assigned negative weights in these models (Figure S3). Including maternal telomere length as an additional covariate did not impact these results (Table S5). When restricting to paired samples, none of our mixture groups influenced maternal telomere length (Table 3) and PFUdA, PFOA, and BDE-99 were all assigned similar positive weights in the overall mixture model (Figure S4).

In quantile g-computation models stratified by infant sex, a one quartile increase in the overall mixture was associated with a 0.04 (95% CI = -0.03, 0.12) and 0.11 (95% CI = 0.00, 0.23) increase in male newborn telomere length and maternal telomere length among women carrying male fetuses, respectively (Table 4). Among males, PFNA was assigned the largest positive weight in the newborn telomere length overall mixture model (Figure S5) and BDE-99 was assigned the largest positive weight in the maternal telomere length overall mixture model (Figure S6). When analyses were restricted to females, no associations were observed (Table 4).

**Table 4:** Quantile g-computation estimates and 95% confidence intervals for the change in newborn and maternal telomere length (T/S ratio) for a one quartile increase within the overall mixture stratified by infant sex

	**Male**			**Female**		
	**N**	**β**	**95% CI**	**N**	**β**	**95% CI**
**Newborn Telomere Length (***N ***= 291)**						
Overall^a^	145	0.04	(-0.03, 0.12)	146	0.01	(-0.07, 0.09)
PFAS^b^	145	0.01	(-0.05, 0.07)	146	0.00	(-0.07, 0.07)
PBDEs^c^	145	0.03	(-0.02, 0.07)	146	0.02	(-0.03, 0.08)
**Maternal Telomere Length (***N ***= 109)**						
Overall^a^	52	0.11	(0.00, 0.23)	57	-0.01	(-0.09, 0.07)
PFAS^b^	52	0.14	(0.01, 0.27)	57	0.03	(-0.03, 0.09)
PBDEs^c^	52	-0.02	(-0.08, 0.05)	57	-0.04	(-0.09, 0.02)

*Abbreviations*: *CI* Confidence interval

Adjusted for maternal age, race/ethnicity, education, parity, and pre-pregnancy body mass index

*Note*: Beta estimates are interpreted as the effect on telomere length of increasing every exposure in the mixture by one quantile. There are 326 pregnant participants, of which there are 76 maternal–fetal unit pairs. Infant sex was missing for one participant

^a^Adjusted for maternal age, race/ethnicity, education, parity, and pre-pregnancy body mass index

^b^Mixture effect is for only PFAS, adjusted for maternal age, race/ethnicity, education, parity, pre-pregnancy body mass index, and PBDEs

^c^Mixture effect is for only PBDEs, adjusted for maternal age, race/ethnicity, education, parity, pre-pregnancy body mass index, and PFAS

Results from individual linear regression models adjusted for maternal age, race/ethnicity, parity, pre-pregnancy BMI and education, showed an IQR increase in BDE-47 and BDE-99 were associated with a 0.03 (95% CI = 0.00, 0.07) and 0.03 (95% CI = -0.01, 0.06) increase in newborn telomere length, respectively (Table 5). No individual PFAS were associated with newborn telomere length. In models which included maternal telomere length as the outcome of interest, an IQR increase in PFNA (β = 0.03, 95% CI = -0.01, 0.07), PFOA (β = 0.05, 95% CI = 0.01, 0.10), and PFOS (β = 0.03, 95% CI = -0.01, 0.08) were modestly associated with increasing maternal telomere length. No PBDEs were associated with maternal telomere length after adjusting for covariates (Table 5).

**Table 5:** Adjusted linear regression coefficients and 95% confidence intervals for newborn and maternal telomere length (T/S ratio) with an interquartile range increase in second trimester PFAS (ng/mL) and PBDE (ng/g lipid) concentrations in maternal serum

	**Newborn Telomere Length (***N ***= 292)**		**Maternal Telomere Length (***N ***= 110)**	
	**Beta**	**95% CI**	**Beta**	**95% CI**
**Per- and poly-fluoroalkyl substances**				
PFNA	0.01	(-0.03, 0.04)	0.03	(-0.01, 0.07)
PFOA	0.01	(-0.03, 0.06)	0.05	(0.01, 0.10)
PFOS	-0.01	(-0.05, 0.03)	0.03	(-0.01, 0.08)
PFHxS	-0.01	(-0.05, 0.04)	0.04	(-0.02, 0.09)
Me-PFOSA-AcOH	0.00	(-0.03, 0.03)	0.01	(-0.02, 0.04)
PFDeA	0.01	(-0.03, 0.04)	0.03	(-0.02, 0.07)
PFUdA	0.00	(-0.05, 0.04)	0.04	(-0.03, 0.1)
**Polybrominated diphenyl ethers**				
BDE-47	0.03	(0.00, 0.07)	0.01	(-0.02, 0.04)
BDE-99	0.03	(-0.01, 0.06)	0.01	(-0.03, 0.04)
BDE-153	0.00	(-0.03, 0.04)	-0.02	(-0.06, 0.01)
BDE-100	0.02	(-0.01, 0.06)	-0.01	(-0.04, 0.03)

*Abbreviations*: *CI* Confidence interval

Adjusted for maternal age, race/ethnicity, education, parity, and pre-pregnancy body mass index

Note: There are 326 pregnant participants, of which there are 76 maternal–fetal unit pairs

## Discussion

In a demographically diverse cohort of pregnant women in the San Francisco Bay Area, we found that prenatal exposure to PFAS and PBDEs were modestly associated with longer newborn telomere length. Associations were greater in magnitude when restricting to maternal–fetal unit paired samples. Associations between increasing concentrations of PFAS or PBDEs and maternal telomere length were smaller and had large confidence intervals. The mixture methods applied in this study were consistent with findings from traditional linear regression models, particularly with respect to PBDEs and newborn telomere length. This is the first study to examine the joint effects of PFAS and PBDEs on telomere length and our results provide important insights into the health effects of cumulative exposure to environmental chemicals, as no individuals are exposed to a single chemical alone.

In addition to examining the joint effects of PFAS and PBDEs combined, we also split them into subgroups and examined mixture effects of PFAS alone and PBDEs alone. Among mixture subgroups, we found that exposure to PBDEs was associated with longer newborn telomere length, with BDE-47 and BDE-99 assigned the largest positive weights. The findings from our mixture analyses and linear regression models were consistent and demonstrated BDE-47 and BDE-99 were positively associated with newborn telomere length. One prior study of pregnant women in San Francisco found that increasing concentrations of BDE-153 were associated with increasing newborn telomere length [26]. However, the sum of BDE-47, BDE-99, BDE-100, and BDE-153 was not associated with newborn telomere length in that study population [26]. Differences could be a result of sample size, as our study was larger, or due to the statistical approaches used, as the quantile g-computation approach applied here allows for heterogeneity in the direction of effect among chemicals included in the exposure mixture.

We observed that increasing PFAS concentrations were modestly associated with increasing maternal telomere length in our main analysis, although confidence intervals were wide. Furthermore, in our analysis restricted to maternal–fetal unit paired samples, effect estimates were markedly increased and PFAS were strongly associated with longer newborn telomeres. However, this association was not observed in the larger analytic sample. One possibility for this is that there is less interhuman variability within the paired sample analysis, as newborn telomere length can be, in part, inherited from parental telomere length [42]. These differences could also be due to selection bias, as our subsample of maternal–fetal unit pairs was somewhat higher educated and had a higher percentage of white participants relative to the larger study population. However, we previously observed no differences in newborn telomere length across racial and ethnic groups [18] and the distribution of PFAS, PBDEs, and telomere length were similar among the maternal–fetal unit pairs compared to the larger analytic samples. We also cannot rule out the possibility that the findings are due to chance given the relatively small sample size (*N *= 76) for the matched maternal–fetal unit pairs. Nonetheless, these findings are consistent with a prior study in Belgium, in which a positive association between PFHxS and leukocyte telomere length was observed among adults in the Flemish Environmental Health Study [27]. In contrast, PFOA and PFOS were inversely associated with leukocyte telomere length in that study [27]. In a birth cohort in Shanghai, increasing PFOS and PFDA were associated with decreasing leukocyte telomere length in newborms [43]. However, when stratifying by infant sex in that study, these inverse associations persisted among female newborns only. Among male newborns, all PFAS were associated with a non-significant increase in leukocyte telomere length [43], which is consistent with results from our sensitivity analysis indicating that PFAS are positively associated with maternal telomere length among male newborns. Taken together, these findings underscore the importance of using environmental mixture methods, such as quantile g-computation, which account for multiple chemical exposures and allow exposure-outcome relationships to go in opposing directions.

A novel aspect of our study was that we examined how environmental chemicals influenced maternal telomere length. Telomere length is strongly attributed to genetic factors [44]. It is possible that maternal telomere length could mediate the relationship between environmental exposures and telomere length at birth. In our study, we found that PFAS and PBDEs were not strongly associated with maternal telomere length. Furthermore, adjusting for maternal telomere length in our sensitivity analysis restricted to maternal–fetal unit pairs did not substantially impact effect estimates for newborn telomere length. Our findings contribute to a growing body of literature exploring the effects of environmental chemicals on matched maternal–fetal pairs. Among 169 mother–child pairs in Argentina, increasing concentrations of arsenic were associated with shorter maternal, but not newborn, telomeres [23]. In contrast, lead was inversely associated with newborn telomere length only [23]. Another study of pregnant women in Boston observed that doubling the concentration of certain phenols was associated with shorter newborn telomere length while certain phthalates were associated with longer newborn telomeres [45]. No associations between phenols, phthalates, and maternal telomere length were observed in that study [45]. These conflicting results could, in part, indicate that newborn telomere length are more sensitive to environmental exposures in utero. In contrast, maternal telomere length may be influenced by multiple exposome effects across the lifespan, which may exert more influence on telomere length than prenatal exposures.

Our study has several notable strengths. CIOB is a demographically diverse study population and our results provide important information on the health effects of understudied populations who are often exposed to multiple environmental chemicals. Additionally, we used quantile g-computation to examine the joint effects of exposures to multiple PFAS and PBDEs. This method allows for individual exposures to have either positive or negative relationships with the outcome, which is important given the bidirectional associations observed in our linear regression models and in previous work [27]. This statistical approach is also an advancement over other mixture approaches, such as Weighted Quantile Sum (WQS) that assume directional homogeneity [46]. Furthermore, we observed few associations in our traditional linear regression analyses that examined each chemical individually. Our mixture analyses elucidated relationships that otherwise would have been missed. We also acknowledge analytical limitations. Our sample size was modest which reduced our statistical power. However, quantile g-computation has been shown to produce unbiased effect estimates in small sample sizes [29]. Information on paternal age and paternal telomere length was also unavailable in our study population and both of these factors may influence telomere length at birth [16, 47]. Lastly, as with all observational studies, our results may be subject to residual confounding.

Our results indicate that prenatal exposure to PFAS and PBDE mixtures is modestly associated with longer telomere length among newborns. In our study, these associations were observed only when restricting to maternal–fetal unit pairs. This has implications for disease risk across the life course, as newborn telomere length is predictive of telomere length later in life [11] and telomere lengthening has been associated with increasing cancer risk later in adulthood [48]. Future studies are needed to elucidate the long term health effects of environmental exposure-induced telomere lengthening.

## Supplementary Information

**Additional file 1: Table S1**. Distribution of demographic characteristics in the overall Chemicals in Our Bodies cohort (N=509). **Table S2**. Distribution of telomere length (T/S ratio) in whole blood, per- and poly-fluoroalkyl substances (ng/mL), and polybrominated diphenyl ethers (ng/g lipid) in serum among paired samples (N=76). **Figure S1**. Weights representing the proportion of the positive and negative effects in the (a) overall mixture, (b) PFAS and (c) PBDEs in relation to newborn telomere length (N=292). **Figure S2**. Weights representing the proportion of the positive and negative effects in the (a) overall mixture, (b) PFAS and (c) PBDEs in relation to maternal telomere length (N=110). **Table S3**. Quantile g-computation estimates and 95% confidence intervals for the change in newborn and maternal telomere length (T/S ratio) for a one quartile increase within the (a) overall mixture, (b) PFAS and (c) PBDEs additionally adjusting for nativity. **Table S4**. Quantile g-computation estimates and 95% confidence intervals for the association between the overall mixture and newborn and maternal telomere length (T/S ratio) when using varying number of quantiles. **Table S5**. Quantile g-computation estimates and 95% confidence intervals for the change in newborn telomere length (T/S ratio) for a one quartile increase within the (a) overall mixture, (b) PFAS and (c) PBDEs among **paired samples **(N=76), additionally adjusting for maternal telomere length. **Figure S3**. Weights representing the proportion of the positive and negative effects in the (a) overall mixture, (b) PFAS and (c) PBDEs in relation to newborn telomere length among **paired samples** (N=76). **Figure S4**. Weights representing the proportion of the positive and negative effects in the (a) overall mixture, (b) PFAS and (c) PBDEs in relation to maternal telomere length among **paired samples** (N=76). **Figure S5**. Weights representing the proportion of the positive and negative effects in the (a) overall mixture, (b) PFAS and (c) PBDEs in relation to newborn telomere length stratified by infant sex. **Figure S6**. Weights representing the proportion of the positive and negative effects in the (a) overall mixture, (b) PFAS and (c) PBDEs in relation to maternal telomere length stratified by infant sex.

## Data Availability

Per University of California, San Francisco Institutional Review Board approval, the data that support the findings of this study are restricted for transmission to those outside the primary investigative team. Data sharing with investigators outside the team requires IRB approval. Requests may be submitted to the Program on Reproductive Health and the Environment (PRHE).

## Abbreviations

PFAS: Per- and polyfluoroalkyl substances
PBDEs: Polybrominated diphenyl ethers
CIOB: Chemicals in Our Bodies
CI: Confidence interval
DTSC: Department of Toxic Substances Control
PFNA: Perfluorononanoic Acid
PFOA: Perfluorooctanoic Acid
PFHxS: Perfluorohexanesulphonic Acid
PFOS: Perflucorooctane Sulfonic Acid
Me-PFOSA-AcOH: Methyl-perfluorooctane sulfonamide Acetic Acid
PFDeA: Perfluorodecanoic Acid
PFUdA: Perfluoroundecanoic Acid
BDE-47: 2,2′,4,4′-Tetra-bromodiphenyl ether
BDE-99: 2,2′,4,4′,5-Penta-bromodiphenyl ether
BDE-153: 2,2′,4,4′,5,5′-Hexa-bromodiphenyl ether
BDE-100: 2,2′,4,4′,6-Penta-bromodiphenyl ether
MDL: Method detection limit
GC-HRMS: Gas chromatography/ high resolution mass spectrometry
BMI: Body mass index
PCR: Polymerase chain reaction
DAG: Directed acyclic graph
IQR: Interquartile range
WQS: Weighted Quantile Sum
