# Supplementary material for: Mixture effects of prenatal exposure to per- and polyfluoroalkyl substances and polybrominated diphenyl ethers on maternal and newborn telomere length
Source: Environ Health. 2021 Jun 30;20:76. doi: 10.1186/s12940-021-00765-4 (PMC8247076; doi:10.1186/s12940-021-00765-4)
Supplement: Supplementary file 1 — Additional file 1: Table S1. Distribution of demographic characteristics in the overall Chemicals in Our Bodies cohort (N=509). Table S2. Distribution of telomere length (T/S ratio) in whole blood, per- and poly-fluoroalkyl substances (ng/mL), and polybrominated diphenyl ethers (ng/g lipid) in serum among paired samples (N=76). Figure S1. Weights representing the proportion of the positive and negative effects in the (a) overall mixture, (b) PFAS and (c) PBDEs in relation to newborn telomere length (N=292). Figure S2. Weights representing the proportion of the positive and negative effects in the (a) overall mixture, (b) PFAS and (c) PBDEs in relation to maternal telomere length (N=110). Table S3. Quantile g-computation estimates and 95% confidence intervals for the change in newborn and maternal telomere length (T/S ratio) for a one quartile increase within the (a) overall mixture, (b) PFAS and (c) PBDEs additionally adjusting for nativity. Table S4. Quantile g-computation estimates and 95% confidence intervals for the association between the overall mixture and newborn and maternal telomere length (T/S ratio) when using varying number of quantiles. Table S5. Quantile g-computation estimates and 95% confidence intervals for the change in newborn telomere length (T/S ratio) for a one quartile increase within the (a) overall mixture, (b) PFAS and (c) PBDEs among paired samples (N=76), additionally adjusting for maternal telomere length. Figure S3. Weights representing the proportion of the positive and negative effects in the (a) overall mixture, (b) PFAS and (c) PBDEs in relation to newborn telomere length among paired samples (N=76). Figure S4. Weights representing the proportion of the positive and negative effects in the (a) overall mixture, (b) PFAS and (c) PBDEs in relation to maternal telomere length among paired samples (N=76). Figure S5. Weights representing the proportion of the positive and negative effects in the (a) overall mixture, (b) PFAS and (c) PBDE [file 12940_2021_765_MOESM1_ESM.docx]

Table S1. Distribution of demographic characteristics in the overall Chemicals in Our Bodies cohort (N=509).

|  | **N (%) or Mean (SD)** |
| --- | --- |
| Maternal Age at Enrollment, years |  |
| Mean (SD) | 33 (5.4) |
| Missing | 1 (0.2%) |
| Gestational age at delivery |  |
| Mean (SD) | 39 (2.0) |
| Missing | 22 (4.3%) |
| Pre-pregnancy BMI (kg/m^2^) |  |
| Mean (SD) | 26 (5.5) |
| Missing | 38 (7.5%) |
| Maternal Education |  |
| Less than High School | 60 (12 %) |
| High School Degree or Some College | 138 (27 %) |
| College Degree | 116 (23 %) |
| Graduate Degree | 186 (37 %) |
| Missing | 9 (1.8%) |
| Maternal Race/Ethnicity |  |
| Non-Hispanic White | 191 (38 %) |
| Non-Hispanic Black | 37 (7 %) |
| Asian/Pacific Islander | 85 (17 %) |
| Latina | 173 (34 %) |
| Other | 14 (3 %) |
| Missing | 9 (1.8%) |
| Parity |  |
| No Prior Births | 246 (48 %) |
| One or More Prior Births | 254 (50 %) |
| Missing | 9 (1.8%) |

Abbreviations: SD, standard deviation; BMI, body mass index.

Table S2. Distribution of telomere length (T/S ratio) in whole blood, per- and poly-fluoroalkyl substances (ng/mL), and polybrominated diphenyl ethers (ng/g lipid) in serum among paired samples (N=76).

|  | **% Above MDL** | **% Machine Readable** | **Geometric Mean** | **Geometric SD** | **Percentiles** | | | | |
| --- | --- | --- | --- | --- | --- | --- | --- | --- | --- |
|  |  |  |  |  | **5%** | **25%** | **50%** | **75%** | **95%** |
| **Telomere Length** | | | | | | | | | |
| Newborn | 100 | 100 | 1.4 | 1.2 | 1.1 | 1.3 | 1.4 | 1.6 | 1.9 |
| Maternal | 100 | 100 | 1.1 | 1.2 | 0.9 | 1 | 1.1 | 1.1 | 1.3 |
| **Per- and poly-fluoroalkyl substances (PFAS)** | | | | | | | | | |
| PFNA | 98.7 | 100 | 0.3 | 2.5 | 0.1 | 0.2 | 0.29 | 0.43 | 1.06 |
| PFOA | 100 | 100 | 0.8 | 2.4 | 0.2 | 0.5 | 0.7 | 1.3 | 2.4 |
| PFHxS | 100 | 100 | 0.5 | 2.1 | 0.2 | 0.3 | 0.5 | 0.8 | 1.1 |
| PFOS | 100 | 100 | 2.0 | 2.0 | 0.7 | 1.3 | 2.2 | 3.2 | 5.4 |
| Me-PFOSA-AcOH | 96.1 | 98.7 | 0.0 | 2.2 | 0.0 | 0.0 | 0.1 | 0.1 | 0.2 |
| PFDeA | 75.0 | 92.1 | 0.1 | 2.6 | 0.0 | 0.1 | 0.2 | 0.2 | 0.7 |
| PFUdA | 82.89 | 93.4 | 0.1 | 2.5 | 0.0 | 0.1 | 0.1 | 0.2 | 0.4 |
| **Polybrominated diphenyl ethers (PBDEs)** | | | | | | | | | |
| BDE-47 | 98.7 | 100 | 9.1 | 2.3 | 3.0 | 4.7 | 8.4 | 14.3 | 32.9 |
| BDE-99 | 67.1 | 98.7 | 2.9 | 2.2 | 0.9 | 1.7 | 2.9 | 4.4 | 7.8 |
| BDE-153 | 59.2 | 89.5 | 6.9 | 2.9 | 2.1 | 3.5 | 5.2 | 12.5 | 42.6 |
| BDE-100 | 42.1 | 100 | 2.2 | 2.6 | 0.7 | 1.1 | 2.1 | 3.0 | 9.7 |

Note: Geometric mean, geometric SD, and percentile values use the machine read value if it was available. If there was no machine read value, it was replaced with MDL/square root of 2.

Abbreviations: MDL, method detection limit; SD, standard deviation.


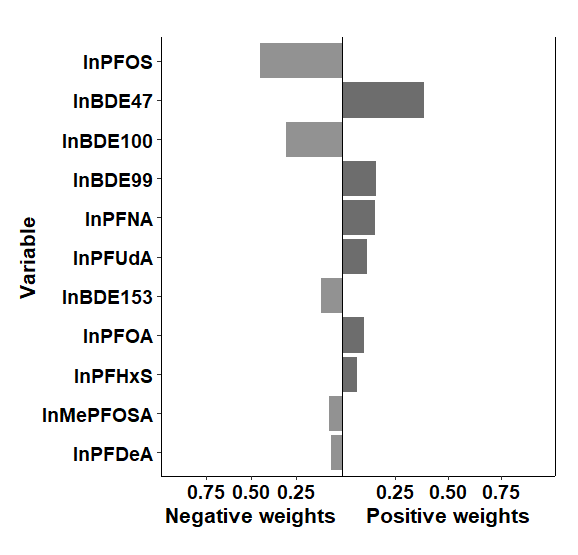

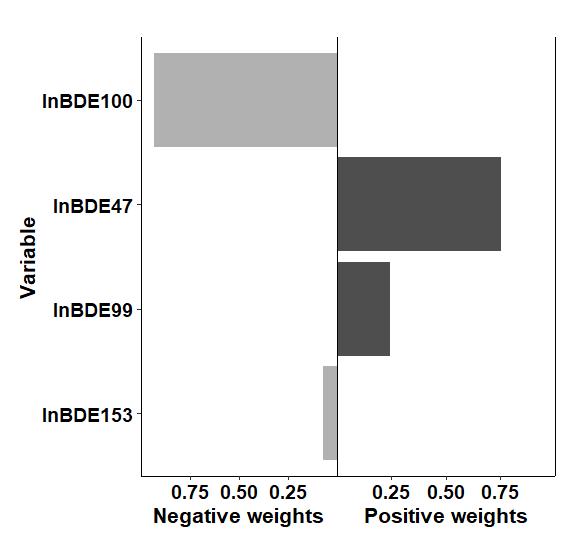

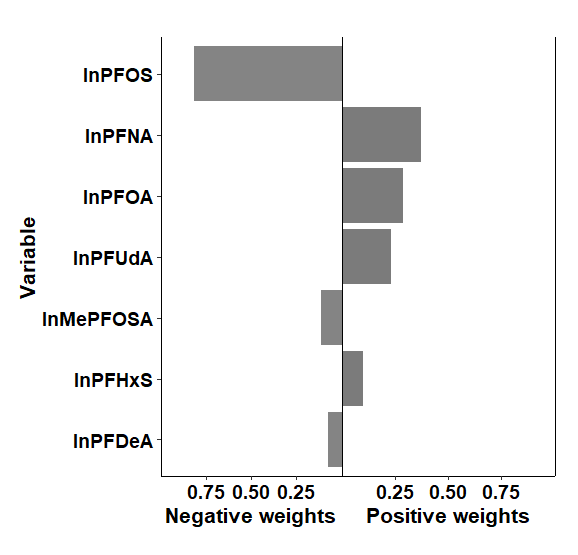


1. (b) (c)

Figure S1. Weights representing the proportion of the positive and negative effects in the (a) overall mixture, (b) PFAS and (c) PBDEs in relation to newborn telomere length (N=292).

Note: Negative and positive weights each sum to 1 and correspond to the effect size relative to other effects in the same direction and should not be directly compared.


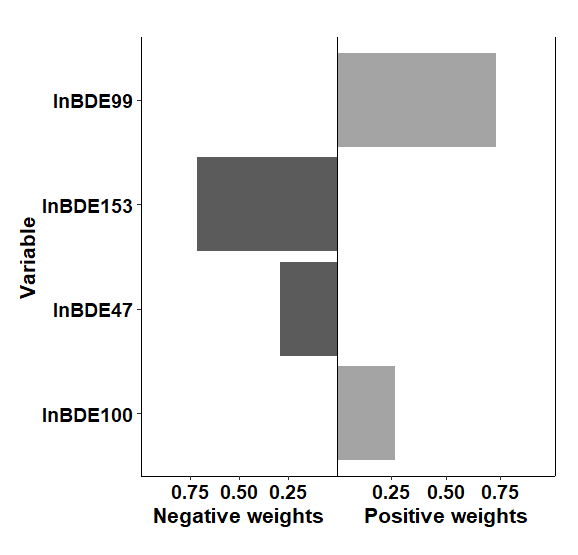

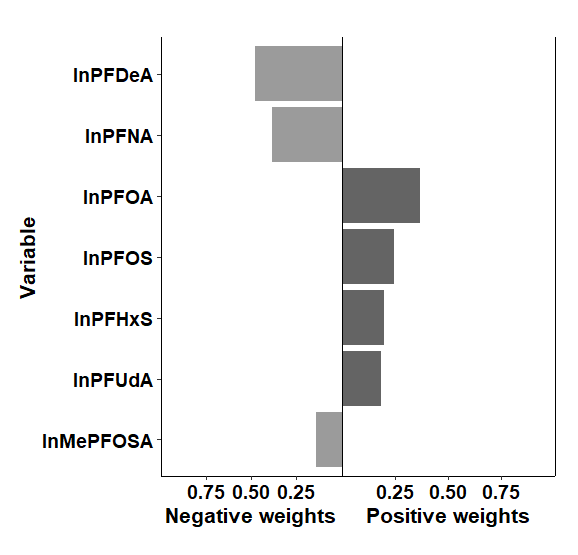

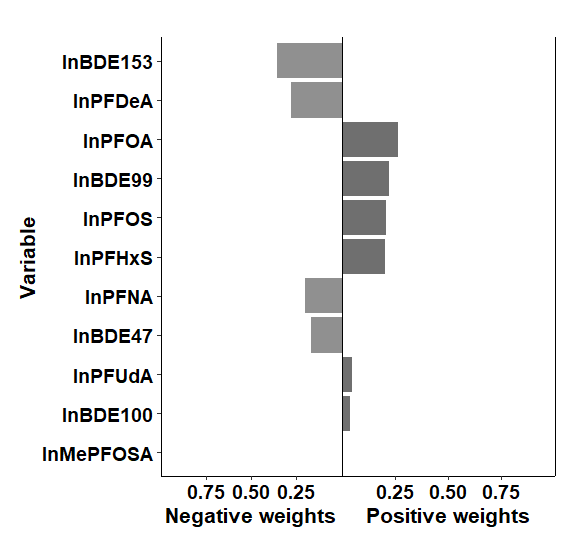


1. (b) (c)

Figure S2. Weights representing the proportion of the positive and negative effects in the (a) overall mixture, (b) PFAS and (c) PBDEs in relation to maternal telomere length (N=110).

Note: Negative and positive weights each sum to 1 and correspond to the effect size relative to other effects in the same direction and should not be directly compared.

Table S3. Quantile g-computation estimates and 95% confidence intervals for the change in newborn and maternal telomere length (T/S ratio) for a one quartile increase within the (a) overall mixture, (b) PFAS and (c) PBDEs additionally adjusting for nativity.

|  | **β** | **95% CI** |
| --- | --- | --- |
| **Newborn Telomere Length (N=283)** | | |
| Overall^1^ | 0.02 | (-0.04, 0.08) |
| PFAS^2^ | 0.00 | (-0.05, 0.05) |
| PBDEs^3^ | 0.02 | (-0.01, 0.06) |
| **Maternal Telomere Length (N=110)** | | |
| Overall^1^ | 0.03 | (-0.03, 0.09) |
| PFAS^2^ | 0.04 | (-0.01, 0.09) |
| PBDEs^3^ | -0.02 | (-0.06, 0.02) |

Abbreviations: CI, confidence interval.

Note: Beta estimates are interpreted as the effect on telomere length of increasing every exposure in the mixture by one quantile. There are 326 pregnant participants, of which there are 76 matched maternal-fetal unit pairs. Nativity was missing for nine participant and the total sample size for newborn telomere length was 283 and 110 for maternal telomere length.

**^1^**Adjusted for maternal age, race/ethnicity, education, parity, pre-pregnancy body mass index, and nativity.

^2^Mixture effect is for only PFAS, adjusted for maternal age, race/ethnicity, education, parity, pre-pregnancy body mass index, nativity, PBDEs.

^3^Mixture effect is for only PBDEs, adjusted for maternal age, race/ethnicity, education, parity, pre-pregnancy body mass index, nativity, and PFAS.

Table S4. Quantile g-computation estimates and 95% confidence intervals for the association between the overall mixture and newborn and maternal telomere length (T/S ratio) when using varying number of quantiles.

|  | **Newborn Telomere Length (N=292)** | | **Maternal Telomere Length (N=110)** | |
| --- | --- | --- | --- | --- |
| **# Quantiles** | **β** | **95% CI** | **β** | **95% CI** |
| 2 | 0.10 | (-0.03, 0.22) | 0.00 | (-0.13, 0.13) |
| 4 | 0.03 | (-0.02, 0.08) | 0.02 | (-0.03, 0.07) |
| 6 | 0.02 | (-0.01, 0.06) | 0.02 | (-0.02, 0.05) |
| 8 | 0.02 | (-0.01, 0.04) | 0.01 | (-0.02, 0.04) |
| 10 | 0.01 | (-0.01, 0.04) | 0.01 | (-0.01, 0.03) |
| 12 | 0.01 | (-0.01, 0.03) | 0.01 | (-0.01, 0.03) |
| 14 | 0.01 | (0.00, 0.03) | 0.01 | (-0.01, 0.02) |
| 16 | 0.01 | (0.00, 0.02) | 0.01 | (-0.01, 0.02) |
| 18 | 0.01 | (0.00, 0.02) | 0.01 | (-0.01, 0.02) |
| 20 | 0.01 | (0.00, 0.02) | 0.01 | (-0.01, 0.02) |

Abbreviations: CI, confidence interval.

Adjusted for maternal age, race/ethnicity, education, parity, and pre-pregnancy body mass index.

Note: Beta estimates interpreted as the effect on telomere length of increasing every exposure in the mixture by one quantile. There are 326 pregnant participants, of which there are 76 matched maternal-fetal unit pairs.

Table S5. Quantile g-computation estimates and 95% confidence intervals for the change in newborn telomere length (T/S ratio) for a one quartile increase within the (a) overall mixture, (b) PFAS and (c) PBDEs among **paired samples** (N=76), additionally adjusting for maternal telomere length.

|  | **β** | **95% CI** |
| --- | --- | --- |
| **Newborn Telomere Length** | | |
| Overall^1^ | 0.13 | (0.03, 0.23) |
| PFAS^2^ | 0.08 | (-0.01, 0.17) |
| PBDEs^3^ | 0.07 | (0.00, 0.15) |

Abbreviations: CI, confidence interval.

Note: Beta estimates are interpreted as the effect on telomere length of increasing every exposure in the mixture by one quantile.

**^1^**Adjusted for maternal age, race/ethnicity, education, parity, pre-pregnancy body mass index, and maternal telomere length.

^2^Mixture effect is for only PFAS, adjusted for maternal age, race/ethnicity, education, parity, pre-pregnancy body mass index, PBDEs, and maternal telomere length.

^3^Mixture effect is for only PBDEs, adjusted for maternal age, race/ethnicity, education, parity, pre-pregnancy body mass index, PFAS, and maternal telomere length.


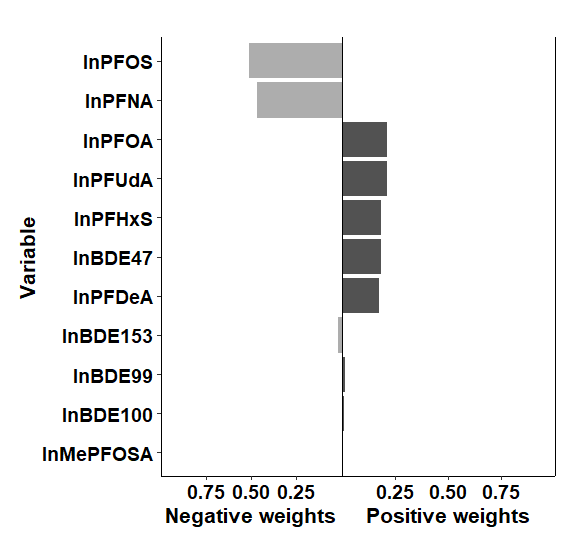

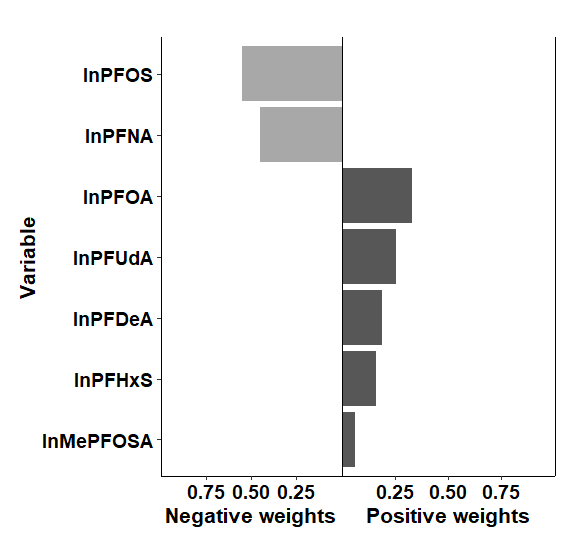

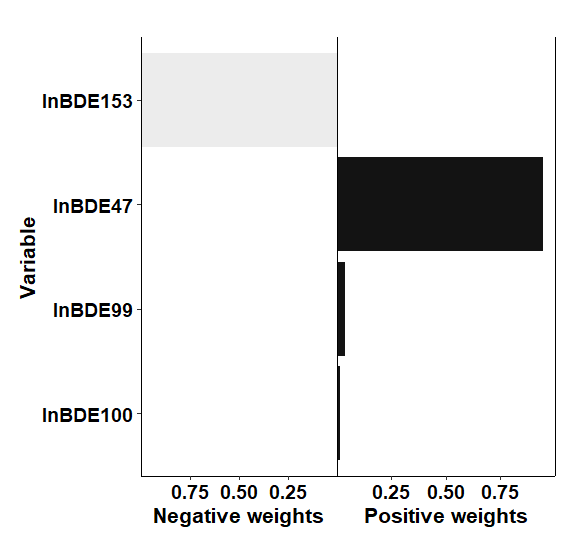


(a) (b) (c)

Figure S3. Weights representing the proportion of the positive and negative effects in the (a) overall mixture, (b) PFAS and (c) PBDEs in relation to newborn telomere length among **paired samples** (N=76).

Note: Negative and positive weights each sum to 1 and correspond to the effect size relative to other effects in the same direction and should not be directly compared.


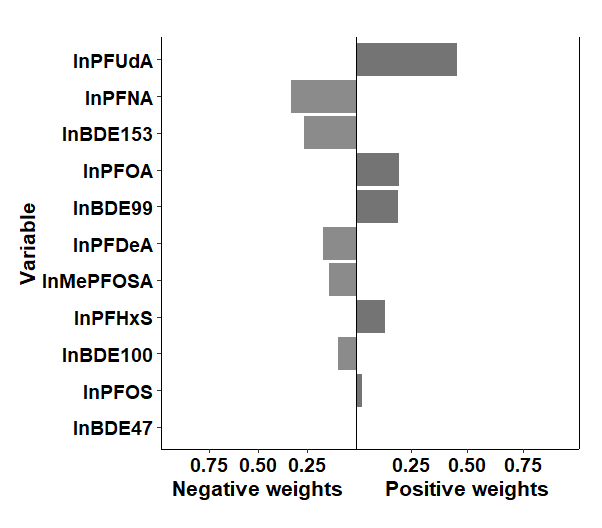

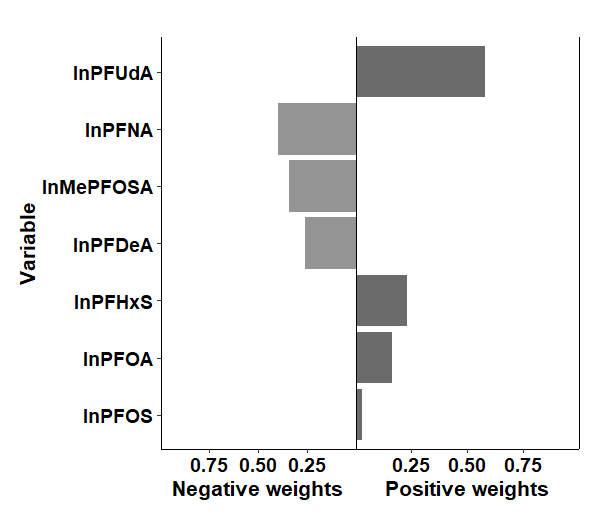

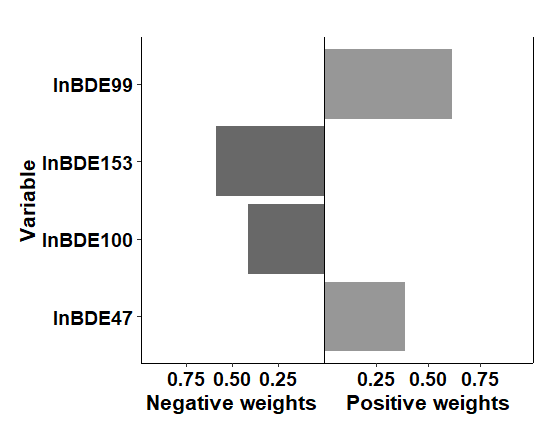


(a) (b) (c)

Figure S4. Weights representing the proportion of the positive and negative effects in the (a) overall mixture, (b) PFAS and (c) PBDEs in relation to maternal telomere length among **paired samples** (N=76).

Note: Negative and positive weights each sum to 1 and correspond to the effect size relative to other effects in the same direction and should not be directly compared.


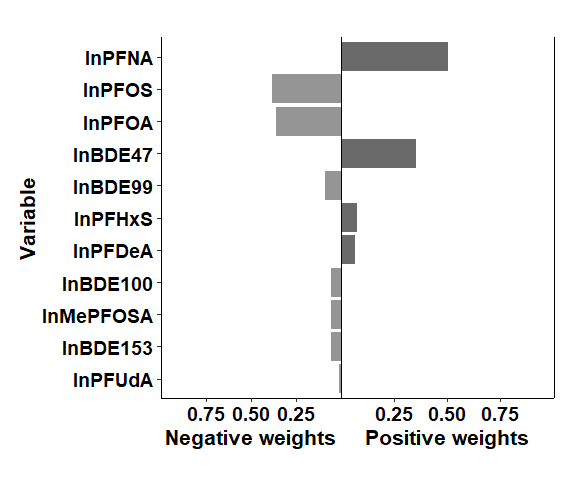

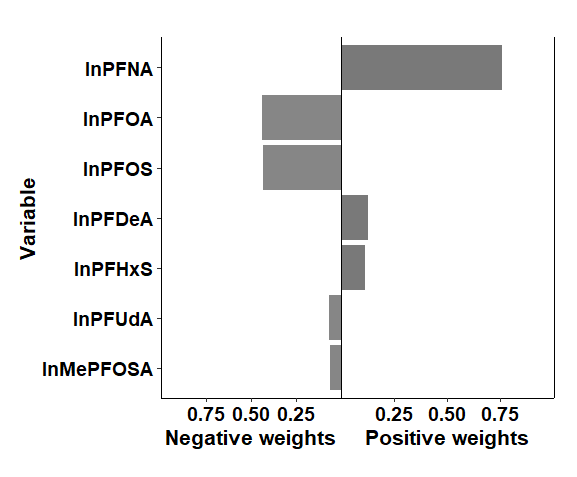

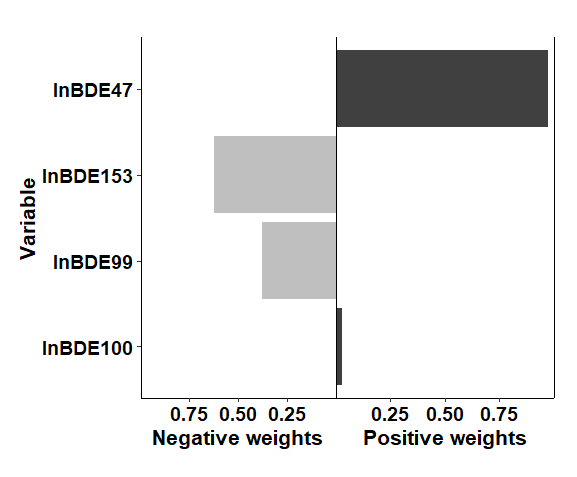


Males: (a) (b) (c)


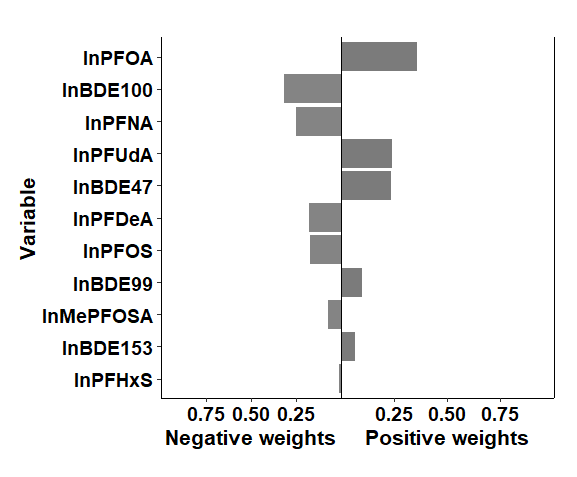

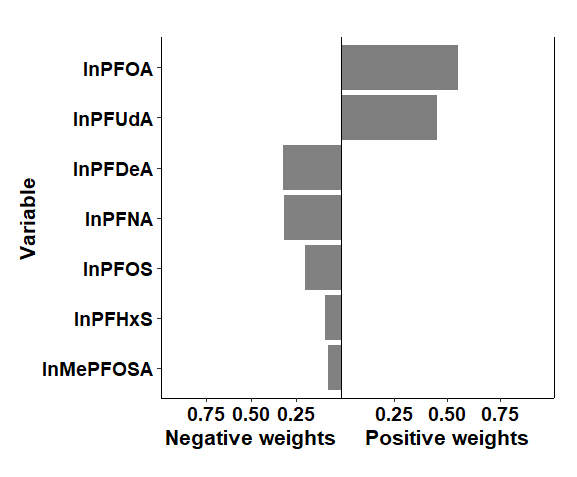

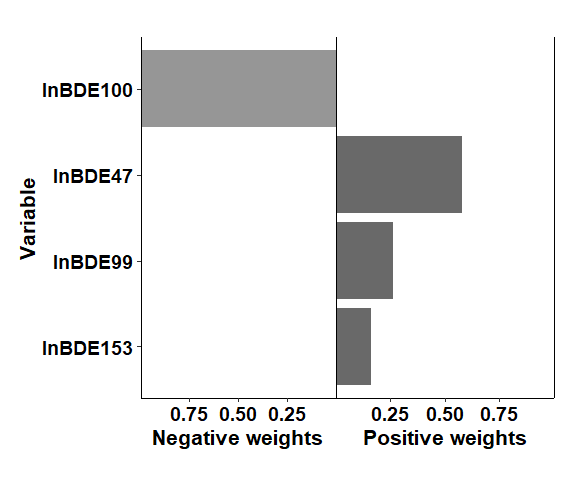


Females: (a) (b) (c)

Figure S5. Weights representing the proportion of the positive and negative effects in the (a) overall mixture, (b) PFAS and (c) PBDEs in relation to newborn telomere length stratified by infant sex.

Note: Negative and positive weights each sum to 1 and correspond to the effect size relative to other effects in the same direction and should not be directly compared.


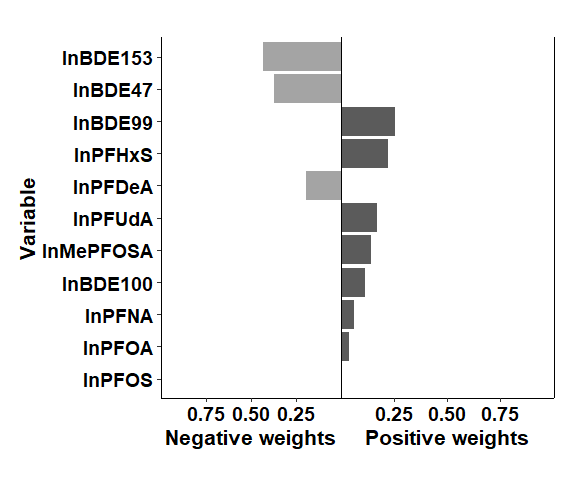

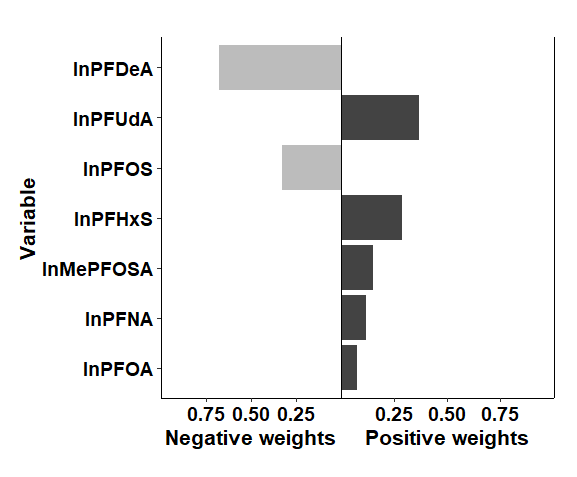

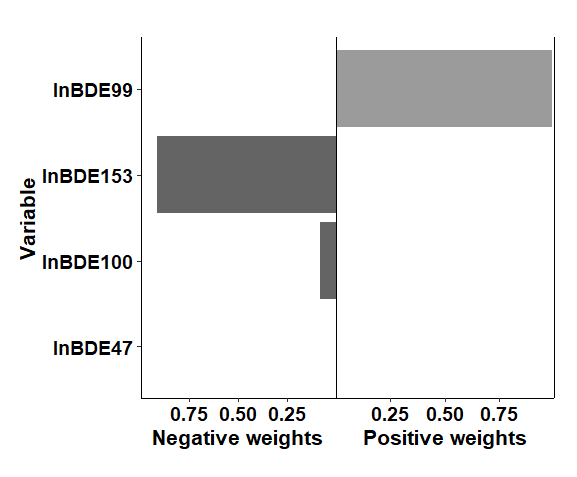


Males: (a) (b) (c)


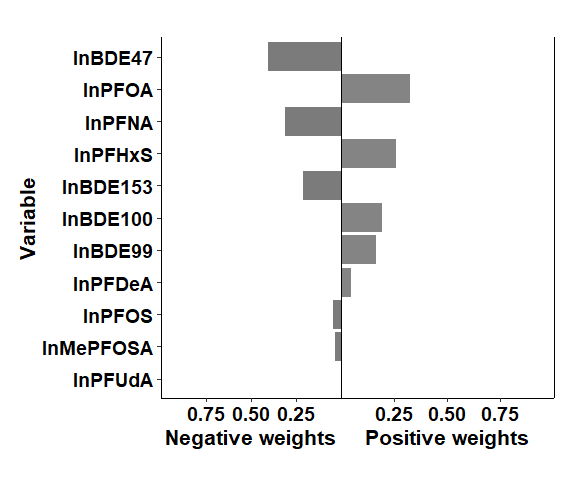

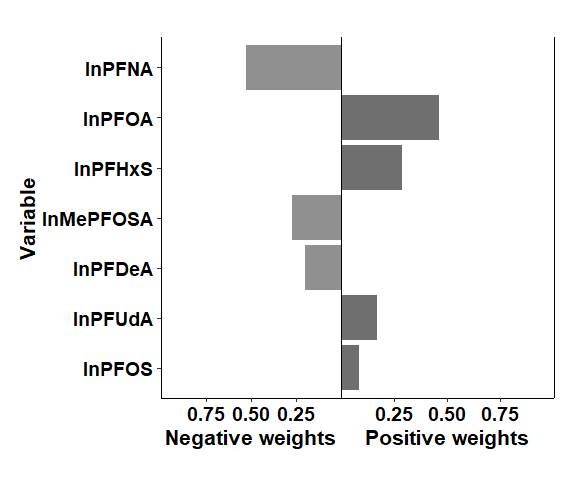

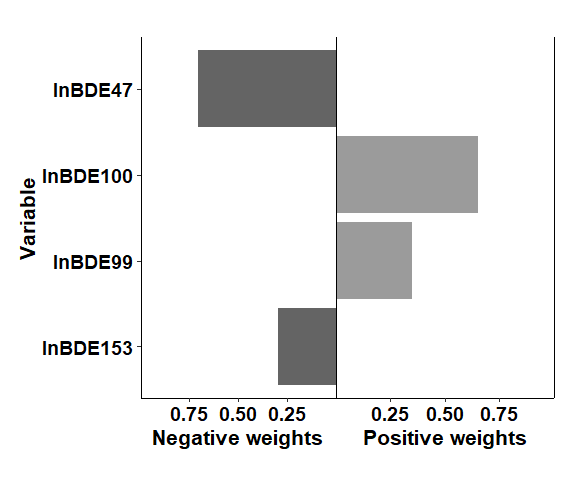


Females: (a) (b) (c)

Figure S6. Weights representing the proportion of the positive and negative effects in the (a) overall mixture, (b) PFAS and (c) PBDEs in relation to maternal telomere length stratified by infant sex.

Note: Negative and positive weights each sum to 1 and correspond to the effect size relative to other effects in the same direction and should not be directly compared.
